# Stress-Induced Cardiomyopathy—Considerations for Diagnosis and Management during the COVID-19 Pandemic

**DOI:** 10.3390/medicina58020192

**Published:** 2022-01-27

**Authors:** Gassan Moady, Shaul Atar

**Affiliations:** 1 Department of Cardiology, Galilee Medical Center, Nahariya 2221006, Israel; shaul.atar@gmail.com; 2 Azrieli Faculty of Medicine, Bar Ilan University, Safed 5290002, Israel

**Keywords:** takotsubo, cardiomyopathy, echocardiography, coronavirus, stress

## Abstract

The novel coronavirus disease 2019 (COVID-19) is associated with several cardiovascular manifestations including myocardial injury, myocarditis, arrhythmia, and pulmonary embolism. Rare cases of stress-induced cardiomyopathy, or takotsubo syndrome have also been reported during the acute infection, and secondary to stress following lockdown and self-isolation. Diagnosis in the setting of the acute infection is challenging since conventional imaging modalities such as transthoracic echocardiography and coronary angiography should be restricted to minimize physician-patient contact until the patients is tested negative for COVID-19. The use of point of care hand-held ultrasound is appropriate for this purpose. The overall course of the disease seems to be similar to takotsubo in the general population. Physicians should be familiar with the clinical presentation, possible complications, and management of takotsubo during COVID-19 outbreak. Here, we review the special considerations in the diagnosis and management of takotsubo syndrome during the current pandemic.

## 1. Introduction

The novel coronavirus disease 2019 (COVID-19) constitutes an ongoing medical challenge through involvement of multiple organs. The disease is caused by the severe acute respiratory syndrome coronavirus 2 (SARS-CoV-2), and it was first identified in December 2019 in the city of Wuhan, Hubei, China [1]. Several COVID-19-related cardiovascular complications have been described, including myocardial injury, myocarditis, arrhythmia, and pulmonary embolism [2,3,4]. In addition, data has accumulated indicating increased morbidity and mortality rates among patients with background cardiovascular disease during the acute infection [5,6,7,8]. Takotsubo syndrome (TTS) is one of the rare cardiovascular manifestations that has also been reported in COVID-19 [9]. TTS, also called “broken heart syndrome” or stress-induced cardiomyopathy, is a type of acute reversible heart failure that mainly affects postmenopausal women. Various physical or emotional stressors, such as fierce argument, public speaking, grief, happiness, major surgery, and various infectious diseases [10,11] typically precedes the syndrome. Clinical presentation often mimics acute coronary syndrome (ACS) with chest pain, ECG and echocardiographic changes, and elevated cardiac biomarkers [12,13]. Key criteria for diagnosis include typical echocardiographic appearance of basal hypercontractility and apical ballooning, and patent coronary arteries without obstructive disease during angiography [14]. Here, we review the special considerations for the diagnosis and management of TTS and the possible impact of the overwhelming stress accompanying its incidence during the current pandemic.

## 2. Takotsubo Syndrome in the Setting of Acute COVID-19

### 2.1. Epidemiology

Similar to other cardiovascular complications, TTS have been reported early with the onset of the COVID-19 pandemic [15,16,17]. In the majority of the reported cases, TTS was with typical echocardiographic appearance of apical ballooning and basal hypercontractility. Late onset [18] and reverse variants (characterized by hypercontractility of the apical region and basal hypokinesia) have also been reported [19,20,21,22,23], and in one case, a patient presented with acute ischemic stroke [24]. According to several echocardiographic studies, the incidence of TTS among infected patients with COVID-19 is around 2–4% [15,16,17]. In the general population, the incidence is about 1–2% among patients presenting with suspected acute coronary syndrome, and it is expected to be higher in elderly women [25,26]. While traditionally TTS predominantly affects women, about one third of COVID-19 related cases were in men [27].

### 2.2. Pathways and Potential Mechanisms

Acute emotional or physical stressors induce an increase in the levels and bioavailability of catecholamines and cortisol in the blood, which mediate several pathways of epicardial coronary spasm, microvascular dysfunction and direct myocyte injury, all are key findings in TTS [28,29,30]. Clues for the essential role of catecholamines in TTS include the high plasma levels in the affected patients, and the induction of TTS-like disease following epinephrine or norepinephrine administration [31,32]. While catecholamines produce positive inotropic effects through Gs-coupling protein, they activate the β_2_-Adreoreceptor differently in cardiac tissue. When high levels of epinephrine (but not epinephrine) are secreted, it triggers the β_2_-adrenoceptor to switch from Gs to Gi coupling. This switch to Gi is dominant in the apical part of cardiac tissue, and it is aimed at limiting the degree of myocardial injury during catecholamine surge induced by the cardiotoxic activation of β_1_- and β_2_-adrenoceptor Gs pathways [33]. This unique pattern of protein coupling results finally in cardiac inhibition in the apical part with concomitant compensatory stimulation of the basal section. The result is the unique apical ballooning appearance observed in takotsubo. Of note, biomarkers of myonecrosis, such as troponin are mildly elevated in TTS except for rare cases of severe disease, whereas natriuretic peptides (NPs), a marker for cardiac wall stress, are typically highly elevated [34,35]. Patients with COVID-19 have high circulating levels of catecholamines secondary to endogenous excretion during the acute stress, and often to exogenous intravenous infusion of adrenaline and noradrenaline used to maintain adequate blood supply in critically ill patients [36]. Other than catecholamines, the hypothalamic–pituitary–adrenal (HPA) axis is also activated in severe COVID-19, which finally results in hypercortisolism and corticotropin suppression [37]. Notably, the relation between high cortisol levels and TTS is not well established yet, since TTS has been described in cases of cortisol excess [38] and in secondary adrenal deficiency [39]. In addition, cumulative evidence does exist regarding the neural spread of the virus, mainly in the cortex and hypothalamus [40]. The release of several inflammatory cytokines such as interleukin-6 and tumor necrosis factor-α in the cytokine storm accompanying severe COVID-19 may also induce catecholamines surge [41]. Probably, the mechanism of TTS during the acute infection is multifactorial and mediated by direct effects of cytokine storm and microvascular dysfunction as seen in other infections. It is not clear, however, why high incidence of TTS has been reported during specific infections other than SARS-CoV-2, particularly influenza virus infection [42,43,44,45,46,47]. Moreover, TTS cases have been reported also following COVID-19 vaccinations [48]. It should be noted that disease-induced stress and catecholamine surge may also aggravate these pathways, and such an overlap in mechanisms seems reasonable. Elevated levels of catecholamine were observed in the aortic root and coronary sinus in patients with COVID-19 and TTS [9].

### 2.3. Diagnostic Considerations

#### 2.3.1. Echocardiography

One of the major limitations in managing patients during the COVID-19 pandemic is the need for minimizing physician–patient contact, at least for the first 1–2 days of patient admission. The current practice during the new Omicron variant outbreak (and perhaps for possible upcoming variants), even with the high percentage of vaccinated patients, is to minimize contact until the patient is tested negative. This need leads to restricted use of several essential imaging modalities, and to the emerging increased use of modified techniques. The use of hand-held point-of-care ultrasound (POCUS) is extremely beneficial for rapid screening of the cardiac structure and function. The yield of POCUS was proved in several cardiovascular conditions including pulmonary embolism, tamponade, myocardial infarction and TTS [49,50,51]. The feasibility and rapid use of POCUS enables general assessment of cardiac condition with minimal exposure to infected patients.

#### 2.3.2. ECG

Common ECG findings in TTS include ST-segment elevation in the precordial leads with no reciprocal changes or Q waves, anterior T wave inversion, and QT-interval prolongation [52,53,54]. It should be noted that in COVID-19, QT prolongation may be a side effect of using proarrhythmic drugs such as quinidine and azithromycin [55,56,57].

#### 2.3.3. Biomarkers

One of the laboratory characteristics of TTS is the discordance in elevation of troponin and N-terminal pro B-type natriuretic peptide (NT-proBNP) levels. Since TTS is not associated with myonecrosis, the elevation in troponin is usually mild unless in severe forms of TTS, while NT-proBNP is significantly elevated reflecting increased wall stress [34,35]. The high NT-proBNP/troponin ratio should be used cautiously in COVID-19 patients for the purpose of TTS diagnosis, since high values of these biomarkers are already increased in patients tested positive for COVID-19.

#### 2.3.4. Coronary Angiography and Cardiac Computed Tomography

**Figure 1:**
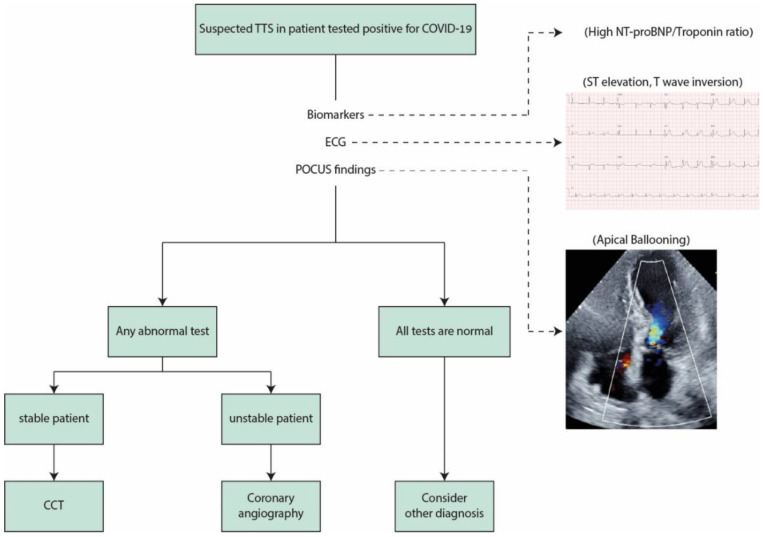
TTS diagnostic algorithm during COVID-19. TTS: takotsubo syndrome; COVID-19: coronavirus disease 2019; POCUS: point-of-care cardiac ultrasound; CCT: coronary computed tomography.

The demonstration of patent coronaries without obstructive disease (particularly in the left anterior descending coronary artery) is essential for TTS diagnosis. According to the current reported cases of COVID-19 related TTS, angiography was performed in about 50% of the cases [27]. This finding is probably a consequence of the recommendation for restricting the contact with patients. The use of other modalities such as computed tomography is a reliable alternative for this purpose. In stable patients with preserved global left ventricular function and high suspicion for TTS according to clinical course, ECG, and echocardiography, exclusion of obstructive coronary artery disease using cardiac computed tomography is recommended in order to reduce the risk of contamination. In unstable patients, coronary angiography is mandatory. A simple algorithm for TTS diagnosis during the COVID-19 pandemic is given in Figure 1.

In case of suspected TTS in patients tested positive for COVID-19, basic tests are recommended. Typical biomarker profile is mild elevation in troponin with excessive NT-proBNP elevation reflecting minor myonecrosis and high cardiac wall stress. The use of POCUS is useful and helps to identify typical wall motion abnormality and exclude other conditions. If all these tests are within normal limits, TTS is unlikely. When the diagnosis is very likely based on the aforementioned tests, and the patient is in stable condition, exclusion of obstructive coronary artery disease using CCT is required. In unstable patients, invasive coronary angiography is the recommended next step.

### 2.4. Clinical Course and Outcomes

Based on the current available data, 80% of patients with COVID-19 related TTS experienced complete recovery. Nearly 60% of patients received inotropic or ventilatory support, whereas mechanical circulatory support (V-V ECMO) was provided only in one patient [27,57]. Thromboembolic events occurred in 2 cases [24,58] and arrhythmia in another 2 cases [59,60]. It should be noted that thromboembolic (venous and arterial) events have been reported in COVID-19 patients regardless to the occurrence of TTS, particularly in severe cases. In one large registry, major arterial or venous thromboembolic events occurred in 35.3% of COVID-19 patients admitted to intensive care unit (ICU) and in 2.6% of hospitalized non-ICU patients, while symptomatic venous thromboembolism was documented in 27% and 2.2% of ICU and non-ICU patients, respectively [61]. Table 1 summarizes the outcomes of selected cases of TTS in COVID-19 patients [18,19,20,21,22,23,24,57,58,59,60,62,63,64,65,66,67,68,69,70,71,72,73,74,75,76,77,78,79].

### 2.5. Treatment

The treatment of COVID-19 related TTS should be based on treating the infectious disease. Generally, the treatment of TTS is conservative with focus on mental and physical stress relieve. When left ventricular dysfunction is present, beta-blockers and Angiotensin-converting enzyme (ACE) inhibitor are recommended and associated with improved survival [80,81]. Caution is needed when there is left ventricular outflow tract obstruction since inotropic agents are contraindicated in these cases while beta-blockers are beneficial in reducing the obstruction [17]. Antiplatelet therapy is not routinely recommended and may be associated with increased mortality [82]. Despite the role of catecholamine in the pathogenesis of TTS, there is no consensus that beta-blockers use is associated with decrease in TTS recurrence [22]. It should be emphasized that although TTS course is generally benign, lethal complications such as cardiogenic shock, malignant ventricular arrhythmia, and thromboembolic events may occur, and the overall prognosis is comparable to that of ACS [12,83].

### 2.6. Stress-Induced Takotsubo during Lockdown and Self Isolation

One of the major impacts of the current pandemic are its psychological and social effects, mainly among elderly. High rates of depression and anxiety during lockdown and self-isolation periods have been reported [84]. The social deprivation, which became a direct consequence of COVID-19, may jeopardize patient adherence to therapy, routine medical check-up and follow-up visits, which in turn aggravates depression and anxiety, creating a vicious cycle. The issue whether TTS incidence was affected by COVID-19 burden was addressed in several studies with inconsistent results [85,86,87]. The difference in the results between the studies may be related to different social status of the study population, length of the study period, and the effect of the pandemic on the particular region. Overall, there appears to be an association between TTS incidence and COVID-19 since this cardiomyopathy is mainly mediated by stress-related pathways [88].

## 3. Prevention

The main potential strategies for TTS prevention during COVID-19 should be focused on limiting stress during this everchanging pandemic. Vaccination against the virus is effective in disease prevention, and it should be encouraged particularly for elderly people and high-risk patients with background comorbidities. It is reasonable to assume that vaccinated patients will experience less stress and anxiety during the pandemic waves. Physicians should be familiar with the possible associations between TTS and COVID-19 since rapid diagnosis and management is essential for avoiding unnecessary medications. Currently, no specific drug is proven to prevent or reduce the incidence of recurrent takotsubo.

## 4. Conclusions

Similar to other cardiovascular problems, the COVID-19 pandemic poses several challenges in TTS management as well. In order to reduce the risk of contact with patients, POCUS and CCT are preferred over standard echocardiography and invasive angiography, respectively. The management of COVID-19 related TTS should not be different from TTS in the general population. The incidence of stress-induced cardiomyopathy is expected to increase in the general population driven by the ongoing social deprivation and depression. Physicians should be familiar with the clinical presentation, possible complications, and management of takotsubo during COVID-19 outbreak.

## Figures and Tables

**Table 1:** Outcomes of patients with TTS in COVID-19.

	Complications	Inotropic Support	Mechanical Ventilation	Outcome
Bottiroli et al. [18]	Shock	Y	Y	Recovery
Faqihi et al. [19]	Shock	Y	Y	Recovery
Nguyen et al. [20]	QT prolongation	N	Y	Recovery
Panchal et al. [21]	Shock	Y	Y	Death
Sala et al. [22]	-	N	N	Recovery
Dabbagh et al. [23]	-	N	N	Recovery
Kariyanna et al. [24]	Shock, acute ischemic stroke	Y	N	Death
Chao et al. [57]	QT prolongation	Y	Y	Recovery
Bernardi et al. [58]	LV thrombi	Y	N	Recovery
Sattar et al. [59]	Atrial fibrillation	N	N	Recovery
Tsao et al. [60]	Ventricular tachycardia	Y	Y	Recovery
Titi et al. [62]	-	Y	Y	Death
Bapat et al. [63]	QT prolongation	Y	Y	Recovery
Bhattacharyya et al. [64]	-	N	N	Recovery
Dave et al. [65]	Shock	Y	Y	Death
Gomez et al. [66]	QT prolongation	Y	Y	Recovery
Torabi et al. [67]	Cardiac tamponade	N	N	Death
Koh MCY et al. [68]	-	N	N	Recovery
Khalid et al. [69]	Shock	Y	Y	Recovery
Manzur-sandoval et al. [70]	QT prolongation	Y	Y	Recovery
Minhas et al. [71]	Shock	Y	N	Recovery
Moderato et al. [72]	QT prolongation	N	N	Recovery
Oyarzabal et al. [73]	-	N	N	Recovery
Pasqualetto et al. [74]	QT prolongation	N	N	Recovery
	-	Y	Y	Death
	-	N	N	Recovery
Van Osch et al. [75]	QT prolongation	N	Y	Recovery
Roca et al. [76]	-	N	N	Recovery
Sang et al. [77]	-	Y	Y	Death
Taza et al. [78]	-	N	N	Recovery
Solano-López et al. [79]	-	N	N	Recovery

## Data Availability

Not applicable.
